# The emerging roles of cerebral pericytes in brain metastasis

**DOI:** 10.1093/neuonc/noag110

**Published:** 2026-05-19

**Authors:** Chanawee Hirunpattarasilp, Herbert B Newton

**Affiliations:** Department of Neurology, University Hospitals Cleveland Medical Center, Cleveland, Ohio, USA; Princess Srisavangavadhana Faculty of Medicine, Chulabhorn Royal Academy, Bangkok, Thailand; Neuro-Oncology Program and Brain Tumor Institute, University Hospitals of Cleveland Medical Center, Seidman Cancer Center, Cleveland, Ohio, USA; Molecular Oncology Program, Case Comprehensive Cancer Center, Cleveland, Ohio, USA

**Keywords:** brain metastasis, pericytes, premetastatic niche, tumor microenvironment

## Abstract

Brain metastases are a prevalent disease found in up to 40% of patients with solid cancers, and are associated with significant morbidity and mortality. Current treatment options for brain metastases are limited, especially because the tumor is protected by the blood-tumor barrier that prevents the penetration of chemotherapeutic agents. Understanding the brain metastatic process and the cells involved is required to discover new treatment approaches. One interesting therapeutic target is mural cells called pericytes, given their importance in maintaining the blood-brain barrier, regulating blood flow, modulating the immune response, and promoting angiogenesis in normal brain physiology. The role of cerebral pericytes in the initiation and maintenance of brain metastases is not well understood. In this review, we discuss the current understanding of the steps in the metastatic process and the roles of cerebral pericytes at each step, especially premetastatic niche formation, cancer cell extravasation, brain micrometastasis formation, and maintenance of macrometastasis and the tumor microenvironment. This study aims to provide a comprehensive understanding of the importance of brain pericytes in brain metastasis formation and maintenance, offer a perspective on the further investigations that are needed, and propose possible clinical implications for the treatment of brain metastases.

## Graphical Abstract

**Graphical Abstract:**
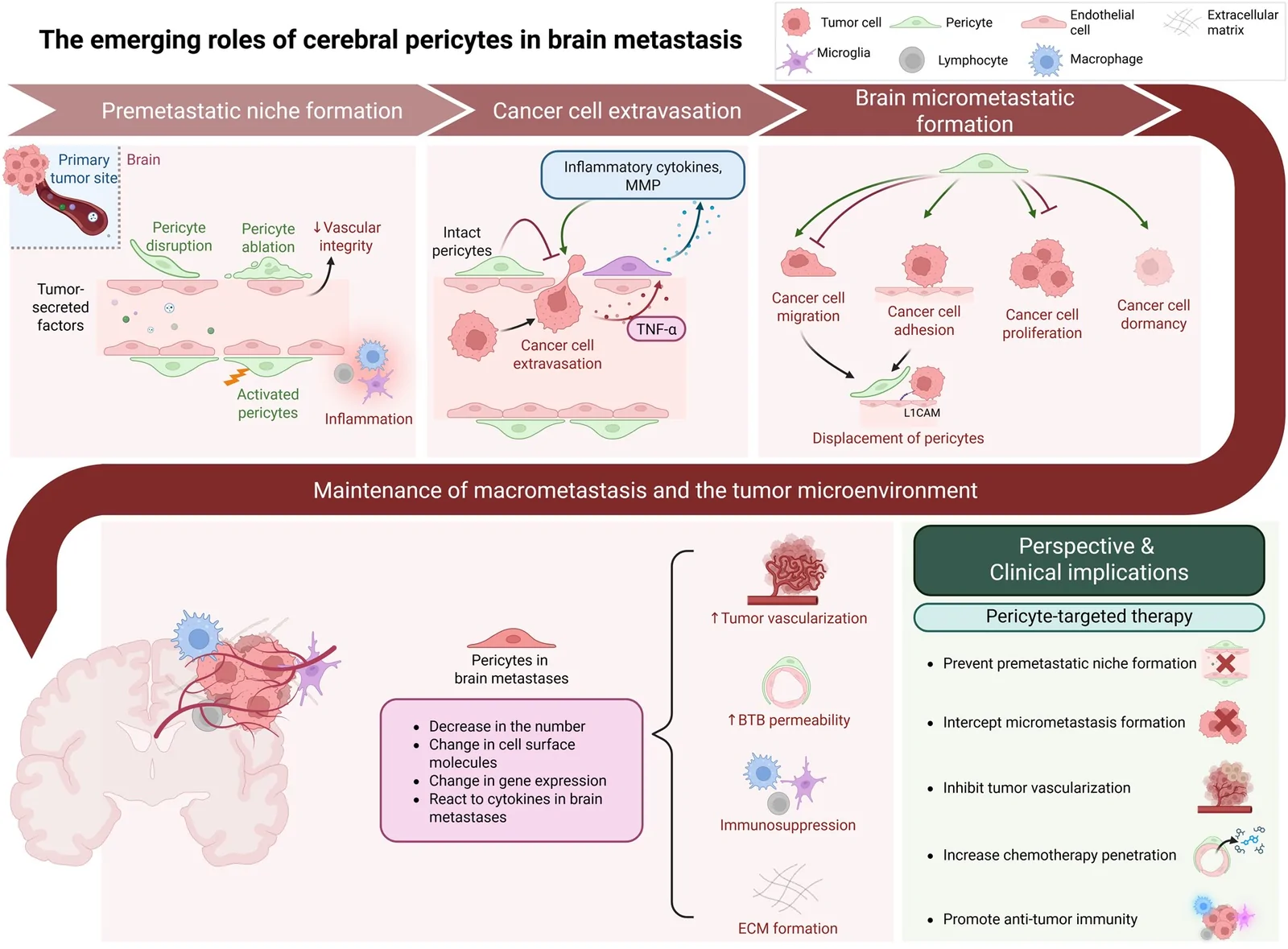

Key Points

- Brain pericytes participate in premetastatic niche formation, extravasation, micrometastasis formation, and maintenance of the tumor microenvironment of brain metastases.
- Pericytes are a potential therapeutic target for brain metastases.

Metastatic brain tumors (BrMs) are a common complication found in 10%-40% of solid cancer patients over their disease course.^1^ It is estimated that there are 70 000-400 000 new BrM cases diagnosed per year in the United States, and BrMs are ten times more prevalent than primary malignant brain tumors.^1^ The most common origins of BrMs are lung cancer (39%-60%), breast cancer (11%-30%), melanoma (6%-10%), colorectal cancer (4%-8%), and renal cancer (2%-6%).^2^^,^^3^ Approximately 2% of patients with solid cancers have BrMs at diagnosis, but the proportion is much higher in those with lung cancer (>10%).^4^

The development of BrMs has a tremendous impact on patients, given the associated neurological manifestations, psychological consequences, and the need to change the oncologic treatment plan for intracranial penetration.^1^ 60%-75% of BrM cases present with neurological symptoms, including seizures, focal neurological deficits, altered mental status, and headaches.^1^ A significant psycho-oncological burden is also found in 60% of BrM patients.^5^ Current treatment options for BrMs are limited because the blood-tumor barrier (BTB) limits the systemic treatment options, and the delicate nature of the brain hinders the ability to perform meaningful local therapies.^6^ Thus, BrM causes significant morbidity and mortality,^7^ with an overall median survival of 7-12 months.^8^ This is further decreased to four months or less in older patients (age ≥ 65 years), regardless of the types of primary cancer.^1^

Brain-metastasizing cancer cells face a unique challenge compared to those spreading elsewhere because the brain has specialized cell types, complex anatomical structures, a distinctive immune landscape, unusual extracellular matrix (ECM) composition, and metabolic constraints, in which neurons utilize most of the oxygen and “outsource” several metabolic tasks to the liver.^9^^,^^10^ The roles of resident neurons, oligodendrocytes, astrocytes, and microglia in BrMs have been extensively studied.^6^ However, there is limited knowledge on the contribution of pericytes, the mural cells on capillaries found at high density in the brain. Given pericytes’ importance in maintaining the blood-brain barrier (BBB), regulating blood flow, modulating local immunity, and promoting angiogenesis in normal brain physiology,^11^^,^^12^ they are gaining attention as key players in BrMs. This review aims to provide a comprehensive understanding of the importance of brain pericytes in BrM formation and maintenance, offer a perspective on the further investigations that are needed, and propose possible clinical implications for BrM treatment.

## Process of Brain Metastasis

**Figure 1.**
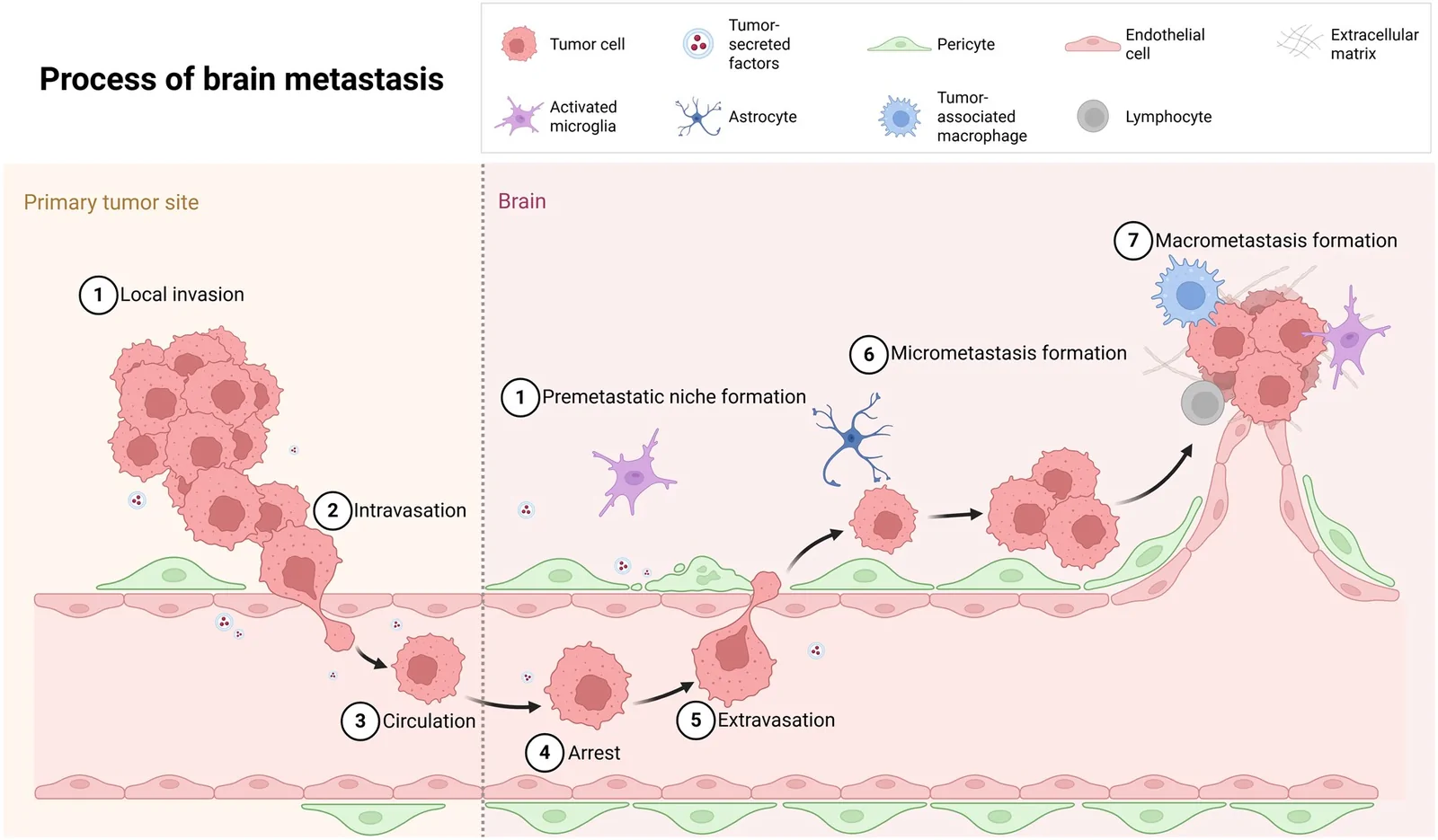
Process of brain metastasis. The pathogenesis of metastatic brain tumors involves multiple steps, including: (1) local invasion and premetastatic niche formation, (2) intravasation, (3) circulation, (4) arrest, (5) extravasation, (6) micrometastasis formation, and (7) macrometastasis formation and maintenance of tumor microenvironment. Created in BioRender. Hirunpattarasilp, C. (2026) https://BioRender.com/oyta8h8.

To understand the role of brain pericytes in BrM initiation and maintenance, it is important to review the metastatic process. The pathogenesis of cancer metastasis involves several key steps (Figure 1), namely: local invasion and premetastatic niche (PMN) formation, intravasation, circulation, arrest, extravasation, micrometastasis formation, and macrometastasis formation, and maintenance of the tumor microenvironment (TME).^13–15^

### Local Invasion and PMN Formation, Intravasation, and Circulation

The first step in the cascade is local invasion, in which cancer cells enter the surrounding normal tissue parenchyma at the primary site.^13^ Subsequently, tumor cells invade blood vessels and enter the lumen in the process called intravasation, passing through pericytes at the primary tumor and endothelial cells. Then, tumor cells disseminate through the circulation.^13^^,^^14^

Simultaneously, tumor cells prime the host microenvironment at secondary sites by establishing PMN, thereby improving access and enhancing early survival.^14^^,^^16^ The key characteristics of PMN include remodeling of the ECM to upregulate pro-adhesive factors, immunosuppression, inflammation, and initiation of angiogenesis.^17^^,^^18^ These changes at secondary sites are mediated by primary tumor-derived molecular components, including secreted factors such as vascular endothelial growth factor (VEGF), placental growth factor (PlGF), and tumor-derived exosomes (containing proteins, RNAs, and DNA fragments).^16^^,^^18^ The PMN formation influences where cancer cells metastasize, thus determining the organotropism of the tumor cells.^18^

### Arrest and Extravasation

Upon arriving at secondary sites, metastasizing cells arrest by adhering to the endothelial cells of capillaries and post-capillary venules.^16^^,^^19^ This process is mediated by the expression of adhesion molecules and reduced blood flow profiles.^16^^,^^20^

Afterward, these cells undergo extravasation, a complex process that involves bypassing the barrier and migrating into the parenchyma. For BrMs, cancer cells must penetrate through the BBB, formed by non-fenestrated endothelial cells connected via tight and adherens junctions, basal lamina, pericytes, and astrocytes.^16^^,^^21^ Extravasation is the rate-limiting step in the BrM cascade,^16^ taking about 2-14 days, which is much longer than extravasation into other organs.^22^ Cancer cells can migrate through the BBB via the paracellular route (moving between endothelial cells) and the transcellular route (migrating through endothelial cells).^23^ Moreover, melanoma cells can migrate by inducing endothelial cell apoptosis.^24^ The extravasation involves ligand-receptor activations, chemokines, proteases, and signaling proteins such as tumor necrosis factor-alpha (TNF-α), interferons, and transforming growth factor-beta (TGF-β).^16^^,^^22^ In addition, matrix metalloproteinases (MMPs) released by cancer cells and cells in the BBB help degrade inter-endothelial junctions and ECM.^25^

### Brain Micrometastasis Formation

Most cancer cells that extravasate fail to form metastases^15^ because they cannot adapt to the microenvironment of the metastatic sites.^13^ Particularly, brain-metastasizing cancer cells face a unique challenge due to the distinct characteristics of the brain microenvironment^9^; however, the prior PMN formation helps lessen this burden on the tumor cells.^16^ The interaction of cancer cells and brain parenchymal cells influences micrometastasis growth. For instance, neuronal activity promotes BrM growth,^26^ while surrounding reactive astrocytes both promote and suppress metastasizing cells.^7^^,^^19^ In addition, micrometastasis formation affects the brain microenvironment by instigating astrogliosis, neuroinflammation, and hyperpermeability of the BBB.^27^ Tumor-derived signals also reprogram microglia to facilitate invasion and colonization.^28^

One key obligation for metastasizing cell survival is maintaining direct contact with blood vessels,^6^^,^^29^ and any cells that leave the perivascular region during the first two weeks die.^22^ The perivascular niche supports tumor cell survival and early growth by providing nutrients, along with controlling cellular dormancy and its reactivation.^6^^,^^30^

Cellular dormancy is a reversible, non-proliferative state in which single cancer cells adapt and survive in new environments.^31^ Dormant metastasizing cells are resistant to therapies and less recognizable to the immune cells.^6^^,^^32^ These cells can last for several years and are associated with relapse of metastasis long after successful primary treatment.^31^^,^^32^ This process of dormancy is, in part, regulated by signaling pathways from endothelial cells and astrocytes.^30^^,^^33^

### Formation of Macrometastasis and TME

Surviving the initial brain defenses does not necessarily translate into the ability to proliferate into a larger metastatic mass. Invading cancer cells need to adapt and convert foreign microenvironments into a more supportive niche to proliferate.^9^^,^^13^ This metastatic colonization counteracts suppression from the secondary sites, such as suppressive gene signaling, restricted angiogenesis, and/or active immune attack through the development of a complex TME.^15^^,^^34^ The formation of TME is governed by the crosstalk between tumor cells and neurovascular units (NVU), brain resident cells, and immune cells.^6^^,^^10^

#### Tumor vascularization and metabolic reprogramming

Modulation in the perivascular niche and reorganization of the microvasculature occur to provide access to nutrients and oxygen.^22^^,^^29^ BrM vascularization is achieved through three mechanisms: (1) angiogenesis, (2) co-option of existing vasculature, and (3) vascular mimicry.^16^^,^^35^^,^^36^ The degree of each pathway depends on the primary tumor type.^22^ Angiogenesis is the growth of new vessels, typically mediated by tumor-secreted VEGF.^22^^,^^37^ However, these new immature BrM vessels are aberrant with dilated/multiple lumens.^38^^,^^39^ Vessel co-option is a non-angiogenic process in which tumor cells seize pre-existing vessels by migrating along the abluminal side of vessels and/or infiltrating into the space between pre-existing vessels, resulting in the incorporation of cerebral vessels.^37^ Displacement of resident pericytes and astrocytes, alterations in endothelial cells, and ECM remodeling are reported in these co-opted vessels,^16^ and co-option depends on adhesion molecules such as the L1 cell adhesion molecule (L1CAM) and β1 integrin.^34^ Vascular mimicry is another form of non-angiogenic neovascularization caused by tumor cells trans-differentiating into vascular endothelial-like cells and forming functional, vascular-like, open-lumen structures.^36^

Despite these vascularization attempts, BrMs still face hypoxia and nutrient limitations due to rapid tumor growth and dysfunctional, abnormal blood vessels.^40^^,^^41^ Thus, metastasizing cells undergo “metabolic reprogramming” to adapt their intracellular metabolism.^40^^,^^41^ Hypoxia activates transcription of genes involved in cancer epithelial-to-mesenchymal transition, glycolysis, angiogenesis, and invasion through hypoxia-inducible factors.^41^ Increased signatures of metabolism using lipid and amino acids and oxidative phosphorylation are evident in brain-colonizing cells.^41^ Metabolic reprogramming also produces lactic acid and promotes acidic TME.^40^ Thus, abnormal vascularization influences metabolic reprogramming of cancer cells.^40^

#### BTB maintenance

Metastasizing cells interact with multiple BBB components to facilitate their progression.^42^ The immature vessels from angiogenesis and modification of co-opted vessels cause the BTB to display heterogeneous permeability, which is “leakier” than the normal BBB.^16^^,^^43^^,^^44^ The BTB is characterized by alterations in pericyte distribution, the absence of astrocyte endfeet, and loss of neuronal connections.^43^ In an animal model of BrMs, >89% of tumor lesions had partial BTB permeability, and there was an increased uptake of paclitaxel and doxorubicin; however, this was <15% compared to peripheral metastasis and does not achieve cytotoxic levels.^45^ Thus, the BTB is not sufficiently or homogeneously permeable to make most drug therapy for BrMs effective.^45^^,^^46^

#### Immunomodulation

Another aspect of the TME is imm­unomodulation to create a heterogeneous, imm­unosuppressive BrM microenvironment characterized by chronic inflammation, overexpression of inhibitory immune checkpoint receptors, exhaustion of T-lymphocytes, and attenuation of NK cells.^28^^,^^47^ Resident microglia display immature, pro-inflammatory phenotypes, while monocyte-derived macrophages assume an M2-like phenotype with anti-inflammatory properties.^28^ BrM-associated neutrophils exhibit an inflammatory response and a high level of programmed death-ligand 1 (PD-L1), an immune checkpoint receptor that inhibits anti-tumor activity.^28^ Unlike in the normal brain, lymphocytes are prevalent in BrM TME and contribute to overall immunosuppression with CD8+ T-cells with an exhausted signature and hyporesponsive CD4+ T-cells.^28^ All of these changes foster an immunosuppressive BrM environment, contributing to BrM growth.

#### ECM formation and development of cancer-associated fibroblasts

Other changes in the TME include increased collagen deposition in the ECM.^48^ Collagen can induce immunosuppressive macrophage and microglial polarization with increased infiltration of pro-tumorigenic macrophages.^49^^,^^50^ There is also development of cancer-associated fibroblasts (CAFs), which are important in ECM modification and immune evasion of cancer cells.^51^ Moreover, CAFs contribute to angiogenesis and release of growth factors to promote tumor cell survival and treatment invasion.^49^

#### BrM growth

The growth patterns of BrMs have been classified into three groups: pushing-type, papillary-type, and diffuse growth patterns.^52^ As defined by Téglási et al,^52^ the pushing-type tumors have a smooth metastasis-parenchymal interface and are found in BrMs of colon and lung origins. In contrast, papillary-type growth tumors, mainly found in BrMs of breast origin, have an undulating tumor-parenchyma interface. Diffuse-type BrMs have a diffuse infiltration of cancer cells into the surrounding brain parenchyma. These patterns of metastatic growth influence the vascularization patterns and ECM content such that pushing-type BrMs assimilate fewer vessels and accumulate more collagen in the periphery near the adjacent brain parenchyma, while papillary-type metastases incorporate more vessels and aggregate collagen in the center.^52^

## Cerebral Pericytes

### Definition and Functions

Pericytes are bump-on-a-log-shaped cells embedded in the basement membrane on the abluminal surface of endothelial cells in the capillaries, post-capillary venules, and terminal arterioles.^11^^,^^53^ Central nervous system (CNS) capillaries have the highest pericyte-to-endothelial cell ratios (1:1-1:3), and up to 80% of capillary surfaces are covered by processes of these cells.^12^ These mural cells have a continuum of phenotypes and are classified according to their locations, morphology, and function^12^: (1) Proximal capillary pericytes. These cells are situated on the proximal branches of arterioles and have circumferential processes that envelop the microvessels. They contain a high level of α-smooth muscle actin (α-SMA) and participate in neurovascular coupling and regulation of cerebral blood flow; (2) Mid-capillary pericytes. These cells have a mesh-like appearance on a more proximal side but adopt a thinner longitudinal process in the more distal capillaries. They are vital in BBB maintenance and small molecule transport; (3) Post-capillary pericytes. These cells, also called stellate pericytes, are found on post-capillary venules, have many slender short branching processes, and participate in the regulation of immune cell entry into the brain parenchyma.^12^ There is no specific pericyte marker, but a combination of several mural cell markers, such as α-SMA, platelet-derived growth factor receptor-beta (PDGFR-β), proteoglycan neuron-glial antigen 2 (NG2), alanyl aminopeptidase (CD13), and desmin, is used to identify pericytes.^11^^,^^12^

Aside from blood flow regulation, BBB maintenance, and immune cell regulation, pericytes are also involved in angiogenesis and vessel stabilization, clearance of toxic and waste molecules, generation of stem cells, and scar formation.^11^^,^^12^ Pericytes work with other cells in the NVU to achieve these functions.^54–56^ The crosstalk between pericytes and endothelial cells via platelet-derived growth factor subunit-B (PDGF-B)/PDGFR-β, TGF-β, VEGF, angiopoietin, and Notch pathways is crucial for angiogenesis, vascular stability, and BBB maintenance (Table 1).^42^^,^^54–67^ Interactions between pericytes and astrocytes via calcium and ephrin type-A receptor 4 signaling pathways, and extracellular glycoproteins contribute to blood flow regulation, BBB maintenance, aquaporin-4 polarization, and glial scar formation.^12^^,^^56^ Pericytes also react to astrocyte-derived cytokines, such as TNF-α and CC chemokine ligands (CCLs).^10^^,^^42^ Moreover, pericytes are involved in microglia proliferation and function by secreting inflammatory and anti-inflammatory cytokines.^12^^,^^56^

**Table 1.** Crosstalk of pericytes and endothelial cells in normal physiology and BrMs^a^

Pathway	Cytokine	Receptor	Functional importance and effect on pericytes and endothelial cells	Signaling alteration in BrMs	Effect in BrMs
PDGF-B pathway^54^ ^,^ ^55^	PDGF-B from endothelial cells^54^ ^,^ ^55^	PDGFR-β on pericytes^54^ ^,^ ^55^	Vascular maturation/stabilization - Pericyte: Promote proliferation, migration, and attachment^54^ - Endothelial cells: Promote tight junction formation^57^	Excess of PDGF-B in TME^42^	- Downregulation of PDGFR-β on pericytes^42^ - Detachment of pericytes from the ECM^42^ - Increase in tumor angiogenesis and vascular permeability^42^
TGF-β pathway^54^ ^,^ ^55^	TGF-β from endothelial cells, pericyte, neurons, and glia^54^ ^,^ ^55^	TGF-β receptors on pericytes and endothelial cells^54^ ^,^ ^55^	Vascular maturation/stabilization and BBB formation - Pericytes: Promote differentiation, migration, and attachment, inhibit pericyte proliferation^54^^,^^55^ - Endothelial cells: Promote tight junction formation and endothelial-to-mesenchymal transition, inhibit proliferation and migration^54^^,^^55^	Excess of TGF-β in TME^58^	- Activation of endothelial-to-mesenchymal transition and formation of unstructured BrM vasculature^59^ - Modify metabolism in pericytes, causing BBB breakdown^60^ - Activation of angiogenesis pathway^59^
VEGF pathway^54^ ^,^ ^55^	VEGF from pericytes^54^ ^,^ ^55^	VEGF receptors on pericytes and endothelial cells^54^ ^,^ ^55^ ^,^ ^61^	Angiogenesis and vascular instability - Pericyte: Promote cell migration, decrease pericyte coverage of blood vessels^61^ - Endothelial cells: Promote growth, survival, and angiogenesis,^55^ decrease tight junction^62^	Excess of VEGF in TME^58^ ^,^ ^63^ ^,^ ^64^	- Ablate pericytes and increase vascular permeability^61^ - Increase angiogenesis^64^
Angiopoietin pathway^54^ ^,^ ^55^	Angiopoietin 1 from pericytes and angiopoietin 2 from endothelial cells^54^ ^,^ ^55^	Tie2 receptors on endothelial cells and pericytes^54^ ^,^ ^55^ ^,^ ^65^	Angiopoietin 1: Vascular maturation/stabilization - Endothelial cells: Stabilize cadherin at cell junctions^55^^,^^62^ Angiopoietin 2: Angiogenesis and vascular instability - Pericytes: Pericyte loss^54^ - Endothelial cells: Antagonize angiopoietin 1 signaling, promote angiogenesis^55^	Excess of Angiopoietin 2 in TME^58^	- Increase angiogenesis^58^ - Promote pericyte loss and increase vascular permeability^66^
NG2 pathway^55^ ^,^ ^67^	NG2 from pericytes^67^	β1 integrin on endothelial cells^67^	Angiogenesis and vascular stabilization - Pericyte: Promote recruitment^67^ - Endothelial cells: Promote motility and angiogenesis^66^	Required for formation and maintenance of BrM vessels^67^	- Inhibiting the NG2 pathway causes poorly functioning tumor vessels^67^

a Abbreviations: BBB, blood-brain barrier; BrMs, metastatic brain tumors; ECM, extracellular matrix; NG2, neuron-glial antigen 2; PDGF-B, platelet-derived growth factor subunit-B; PDGFR-β, platelet-derived growth factor receptor-beta; TGF-β, transforming growth factor-beta; Tie2, tyrosine kinase with immunoglobulin and EGF homology domain 2; TME, tumor microenvironment; VEGF, vascular endothelial growth factor.

Dysfunction of pericytes is related to multiple pathologies, including stroke, epilepsy, Huntington’s disease, Alzheimer’s disease, multiple sclerosis, and Coronavirus disease 2019.^11^^,^^68^^,^^69^ Pericytes also play a key role in glioblastoma.^70^^,^^71^ Increasing evidence demonstrates the importance of pericytes in the formation of BrMs from breast, lung, melanoma, and gastrointestinal origins.^72–76^

Pericytes at primary cancer sites are involved in tumor growth, local invasion, and metastasis; a review of this topic is available elsewhere.^40^ This review focuses on the contribution of cerebral pericytes in the development and maintenance of BrMs. It should be noted that these roles of pericytes in BrMs may vary between different primary tumors.^73^^,^^75^^,^^77^  Table 2 summarizes the experimental models and primary cancer types investigated in each key study, with an appraisal of the evidence for each section.

**Table 2.** Key studies on pericytes in BrMs and appraisal of the evidence

Ref	Model(s)	Primary cancer type(s)	Methodological robustness	Generalizability
Pericyte involvement in PMN formation				
Nairon et al^76^	*In vitro*	Colon	Limited - including only one *in vitro* experiment	Limited - including only one study in colon BrM
Pericyte involvement in cancer cell extravasation				
Fujimoto et al^74^	*In vitro*	Lung	Limited - including only *in vitro* and *in vivo* studies	Limited - no data in human tissue - including two of the most common BrM primaries
Figueira et al^86^	*In vivo*	Breast		
Pericyte involvement in brain micrometastasis formation				
Bagley et al^88^	*In vitro*	Breast	Good - including *in vitro*, *in vivo*, and human tissue studies	Good - Including various BrM primaries
Er et al^87^	*In vitro*, *in vivo*	Lung, breast		
Fujimoto et al^74^	*In vitro*	Lung		
Molnár et al^73^	*In vivo*, *ex vivo human tissue*	Breast, melanoma		
Ujifuku et al^90^	*In vitro*	Lung		
Csonti et al^89^	*In vitro*	Breast		
McErlain et al^91^	*In vitro* (lung pericyte), *in vivo*	Lung		
Noda-Narita et al^77^	*In vitro*	Lung, breast		
Pericyte involvement in formation of brain macrometastasis and TME				
Changes in pericyte number and expression in TME				
Lyle et al^46^	*In vivo*, *ex vivo* human tissue	Breast	Good - including *in vitro*, *in vivo*, human tissue, and bioinformatic studies	Good - Including various BrM primaries
Er et al^87^	*In vitro*, *in vivo*	Lung, breast		
Uzunalli et al^39^	*In vivo*, *ex vivo* human tissue	Lung		
Bejarano et al^75^	*In vivo*, *ex vivo* human tissue, bioinformatic analysis of human cells	Lung, breast, melanoma		
Tumor vascularization and metabolic reprogramming				
Huang et al^67^	*In vivo*	Melanoma	Good - including *in vitro*, *in vivo*, human tissue, and bioinformatic studies	Good - Including various BrM primaries
You et al^94^	*In vitro*, *in vivo*	Melanoma		
Téglási et al^52^	*Ex vivo* human tissue	Lung, breast, colon		
Bejarano et al^75^	*In vivo*, e*x vivo* human tissue, bioinformatic analysis of human cells	Lung, breast, melanoma		
Huang et al^93^	*In vitro*, *in vivo*, *ex vivo* human tissue	Lung		
Wu et al^59^	Bioinformatic analysis of human cells	Lung		
Liu et al^64^	*In vitro*, *in vivo*, *ex vivo* human tissue	Lung		
BTB maintenance				
Lockman et al^45^	*In vivo*	Breast	Good - including *in vitro*, *in vivo*, human tissue, and bioinformatic studies	Good - Including various BrM primaries
Lyle et al^46^	*In vivo*, *ex vivo* human tissue	Breast		
Uzunalli et al^39^	*In vivo*, *ex vivo* human tissue	Lung		
Bejarano et al^75^	*In vivo*, *ex vivo* human tissue, bioinformatic analysis of human cells	Lung, breast, melanoma		
Mu et al^96^	*In vitro*, *in vivo*	Breast		
Immunomodulation				
Bejarano et al^75^	*In vivo*, *ex vivo* human tissue, bioinformatic analysis of human cells	Lung, breast, melanoma	Good - including *in vivo*, human tissue, and bioinformatic studies	Good - Including various BrM primaries
Wu et al^59^	Bioinformatic analysis of human cells	Lung		
Zhu et al^98^	*Ex vivo* human tissue, bioinformatic analysis of human cells	Breast		
ECM formation and development of CAFs				
Téglási et al^52^	*Ex vivo* human tissue	Lung, breast, colon	Good - including *in vivo*, human tissue, and bioinformatic studies	Good - Including various BrM primaries
Akanda et al^51^	*Ex vivo* human tissue	Lung, breast, colon, liver, gastric, melanoma, kidney, thyroid		
Bejarano et al^75^	*In vivo*, *ex vivo* human tissue, bioinformatic analysis of human cells	Lung, breast, melanoma		
Wu et al^59^	Bioinformatic analysis of human cells	Lung		

### Pericyte Involvement in PMN Formation

**Figure 2.**
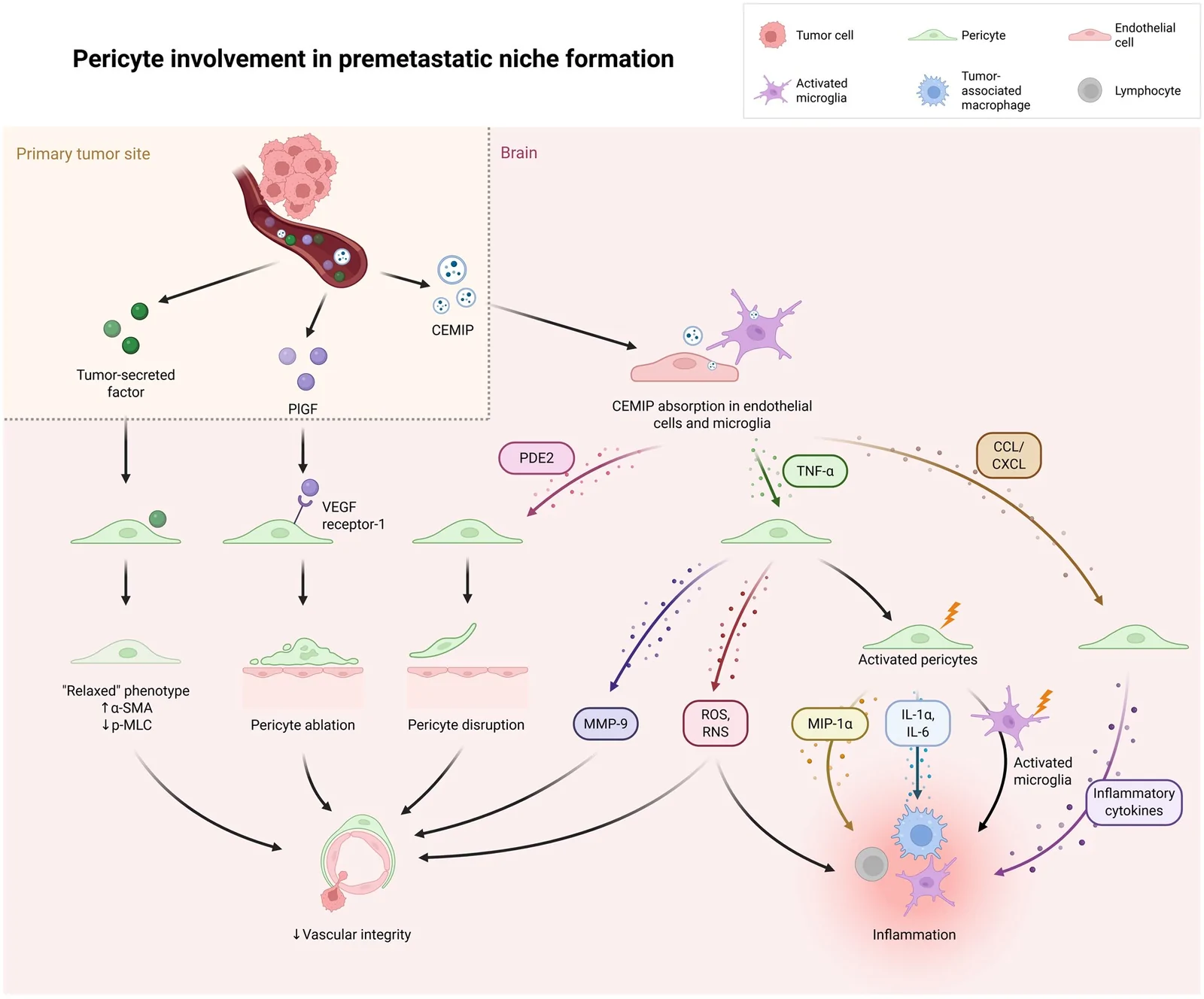
Pericyte involvement in premetastatic niche formation. Cancer cells at primary tumor sites can promote metastases by secreting factors that convert brain pericytes to a “relaxed” phenotype, characterized by increased alpha-smooth muscle actin (α-SMA) expression and reduced phosphorylated myosin light chain (p-MLC), thereby increasing brain vascular permeability. Primary tumor cells can also produce placental growth factor (PlGF) that activates vascular endothelial growth factor (VEGF) receptor-1 on brain pericytes, leading to pericyte ablation and increased cerebral vascular permeability. Tumor-secreted cell migration-inducing and hyaluronan-binding protein (CEMIP) is absorbed by cerebral endothelial cells and microglia. These cells, in turn, release prostaglandin E2 (PGE2), which disrupts pericytes and breaks pericyte-endothelial cell interactions, resulting in decreased vascular integrity. Affected endothelial cells and microglia also produce tumor necrosis factor alpha (TNF-α). TNF-α induces pericyte release of matrix metalloproteinases-9 (MMP-9), further compromising vascular integrity. TNF-α-activated pericytes also produce reactive oxygen species (ROS) and reactive nitrogen species (RNS), which reduce vascular integrity and induce inflammation. Furthermore, TNF-α activates pericytes to promote inflammation by releasing macrophage inflammatory protein (MIP)-1α, interleukin (IL)-1α, and IL-6, and by further activating microglia. CEMIP-activated endothelial cells and microglia also release CC chemokine ligands (CCL) and CXC chemokine ligands (CXCL), which promote inflammatory cytokine release from pericytes, leading to further inflammation. Created in BioRender. Hirunpattarasilp, C. (2026) https://BioRender.com/br1m09g.

Data are scarce on the involvement of cerebral pericytes in PMN formation, with only one study investigating the effect of tumor-secreted factors on pericytes^76^; however, some data can be inferred from other molecular pathways related to PMN formation that might affect pericytes (Figure 2).

*In vitro* exposure of human brain vascular pericytes to conditioned media containing secreted factors from colorectal cancer cell lines, especially the highly metastatic lines, results in a stunted, spherical phenotype with increased α-SMA expression but reduced phosphorylated myosin light chain levels.^76^ This “relaxed” pericyte phenotype with less contractility and interrupted extension corresponds to low pericyte vessel coverage, leading to impaired barrier function and dysregulated leaky vasculature. Thus, this creates a supportive environment for extravasation and invasion of tumor cells.^76^

Elevated levels of PlGF in small cell lung cancer (SCLC) patients were associated with BrM, and downregulation of PlGF in an *in vivo* experiment suppressed the metastasis.^78^ Furthermore, SCLC-derived PlGF broke down tight junctions in brain endothelial cells to promote transendothelial migration of SCLC cells through activation of VEGF receptor-1.^78^ Although the study did not investigate the effect on pericytes, PlGF can activate the VEGF receptor-1 signaling pathway to ablate pericytes from mature retina vessels, leading to increased vascular permeability.^61^ Thus, PlGF signaling could represent another way that pericytes are involved in PMN formation.

In lung and breast cancer patients, the levels of cell migration-inducing and hyaluronan-binding protein (CEMIP) correlated with BrM and survival.^79^ CEMIP was also identified in exosomes secreted by brain-tropic human breast cancer metastatic cells, and application of these CEMIP^+^ exosomes on organotypic brain slice cultures and a mouse model of breast cancer enhanced tumor cell growth and colonization by establishing PMN for BrMs. The mechanism involved was the activation of pro-inflammatory cytokines encoded by *Ptgs2*, *Tnf*, and *Ccl/Cxcl* by endothelial and microglial cells that absorbed these exosomes, leading to vascular remodeling and inflammation in the brain perivascular niche.^79^ Although pericytes were not studied, these involved cytokines are known to affect pericytes. Cyclooxygenase 2 (COX-2), an enzyme encoded by *Ptgs2*, produces prostaglandin E2 (PGE2) as its main product. PGE2 can transform pericytes into a fast-migrating, loosely adherent phenotype, with downregulated N-cadherin at adherens junctions and connexin-43 at gap junctions, and transcriptionally repressed R-Ras expression, which is important for blood vessel stabilization.^80^ As a result, PGE2 causes disruption of pericytes, breakdown of the pericyte-endothelial cell interaction, and destabilization of blood vessels.^80^ This decreased vascular barrier integrity is one of the key characteristics of PMN for cancer invasion.^17^ In addition, cerebral pericytes are the most sensitive cells in the NVU to TNF-α, encoded by *Tnf.*^81^ TNF-α promotes MMP-9 release from cerebral pericytes, causing pericyte migration and BBB breakdown.^82^ Moreover, it increases pericyte expression of inducible nitric oxide synthase and COX-2, leading to the generation of reactive oxygen and nitrogen species.^83^ TNF-α -stimulated pericytes also release inflammatory cytokines such as macrophage inflammatory protein (MIP)-1α, interleukin (IL)-1α, and IL-6, and activate microglia.^81^^,^^84^ These effects of TNF-α lead to inflammation, another hallmark of PMN.^17^ CCL, especially CCL-2, and CXC chemokine ligand (CXCL), encoded by *Ccl/Cxcl*, causes pericytes to secrete more inflammatory mediators and foster inflammation.^85^

### Pericyte Involvement in Cancer Cell Extravasation

Pericytes maintain the BBB, but they can both inhibit and stimulate cancer cell extravasation. Fujimoto et al^74^ performed a transwell experiment of co-culturing human squamous cell lung carcinoma cell lines with each BBB-related cell type and measured endothelial tight junction function and barrier integrity. Only the model with pericytes preserved the BBB integrity during cancer cell introduction, thus suggesting a pericyte role in protecting against cancer cell extravasation.^74^

Systemic injection of aggressive breast cancer cells to develop BrMs caused a significant increase in α-SMA and myosin light chain kinase in pericytes over time during cancer cell extravasation and metastasis formation, along with increased endothelial tight and adherens junctions.^86^ Figueira et al^86^ hypothesized that the resultant mural cell activation with increased contractility restores BBB integrity and prevents further extravasation of breast cancer cells.

In contrast, cytokines released during tumor extravasation could affect pericytes to facilitate the process. Tumor-derived TNF-α^16^^,^^22^^,^^25^ and astrocyte-derived CCL-2^10^^,^^42^ can induce the release of MMP-9 and inflammatory cytokines from pericytes,^82^^,^^84^ thereby compromising BBB integrity and attracting cancer cells.^25^

### Pericyte Involvement in Brain Micrometastasis Formation

**Figure 3.**
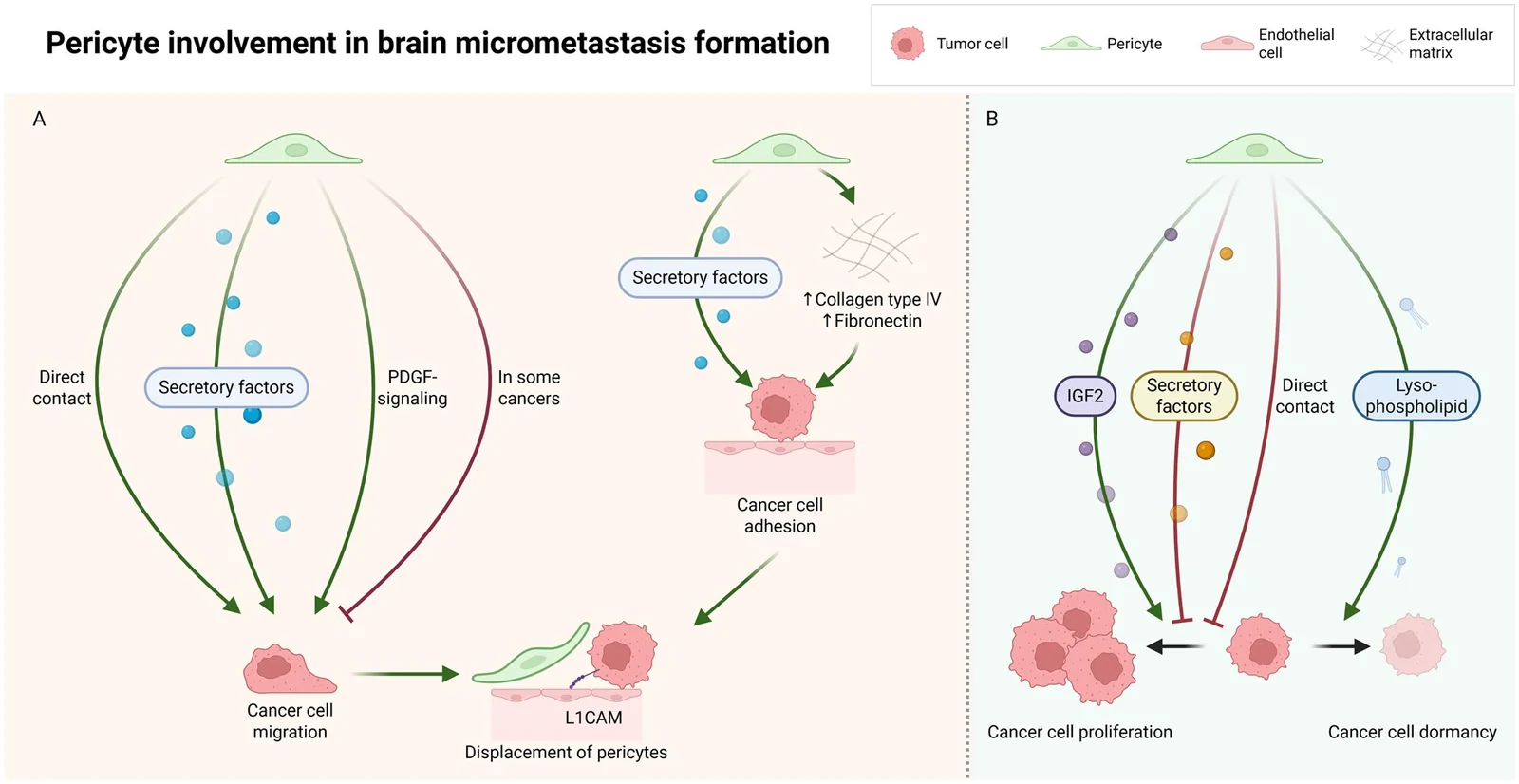
Pericyte involvement in brain micrometastasis formation. (A) Direct contact with pericytes, secretory factors from mural cells, and platelet-derived growth factor (PDGF) signaling enhance cancer cell migration, thus promoting micrometastasis formation. However, some studies found that pericytes can also inhibit tumor migration. Brain pericytes also enhance cancer cell adhesion to blood vessels via secretory factors and extracellular matrix modification (increased collagen type IV and fibronectin). With increased cancer cell migration and tumor cell adhesion, these malignant cells displace pericytes from the perivascular location through competition of the L1 cell adhesion molecule (L1CAM) signaling pathway. (B) Pericytes are shown to affect metastasizing cancer proliferation. Insulin-like growth factor 2 (IGF-2) released by pericytes promotes tumor growth, while other secretory factors and direct pericyte contact can inhibit cancer proliferation. In addition, cerebral pericytes release lysophospholipids that induce cancer cell dormancy, making them resistant to therapies and less recognizable to immune cells. Created in BioRender. Hirunpattarasilp, C. (2026) https://BioRender.com/ymm7qrj.

Pericytes can modulate cancer cell migration and promote their attachment in the perivascular niche, a requirement for these cells to survive and proliferate (Figure 3).^6^^,^^29^ However, these mural cells also compete with the cancer cells for the perivascular location,^87^ and there is conflicting evidence on the proliferative effects of pericytes on BrMs.

In a co-culture system, cerebral pericytes exerted a chemoattractant effect on cultured breast cancer cells, leading to migration and congregation.^73^ This augmentation of cancer cell migration by pericytes was replicated in murine lung cancer cells from direct interaction with pericytes.^77^ Transcriptomic analysis of brain-metastatic lung cancer cells also supports this role of pericytes, showing upregulation of cell-cell and cell-matrix signaling pathways and increased migration when pericytes interact directly with cancer cells.^77^ However, this transcriptomic analysis showed pericyte-induced inhibition of breast cancer cell migration, further highlighting that BrM cancer cells from different origins might react differently to pericytes.^77^ Another possible mechanism of pericyte-enhanced tumor migration is through PDGFR signaling, as blocking PDGFR with the tyrosine kinase inhibitor SU6668 partially inhibited pericyte-enhanced migration of breast cancer cells.^88^ Thus, most of these experiments demonstrate the tumor-supporting role of pericytes by increasing the migration of cancer cells.

In addition, pericytes can strengthen the adhesion of both breast cancer and melanoma cells through the pericyte secretome and the production of high amounts of collagen type IV and fibronectin.^73^ This enhanced cancer cell adhesion by pericytes is demonstrated in another experiment using single-cell force spectroscopy, showing that triple-negative breast adenocarcinoma (particularly the brain tropic variant) adhered more strongly to pericytes compared to endothelial cells.^89^ This long-lasting, strong adhesion with pericytes reflects their support in the cancer cell situation in the perivascular niche, while transitory adhesion to endothelial cells facilitates cancer cell extravasation.^89^

Interestingly, competition between pericytes and brain-metastasizing cancer cells for the perivascular niche has been demonstrated. Brain-metastatic derivatives of lung cancer cells spread along capillaries via tumor L1CAM, ­activating the mechano-transduction effector YAP (Yes-associated protein) signaling, both during initial cancer infiltration and after exiting dormancy.^87^ Because resident pericytes also use L1CAM for perivascular spreading, the lung adenocarcinoma cells compete for L1CAM and displace these mural cells, leading to reduced pericyte coverage on the vessels.^87^ This pericyte-cancer cell competition is further supported by RNA transcriptomic changes in pericytes in an *in vitro* BBB model co-cultured with lung cancer cells, showing a pericyte-lot-specific decrease in expression of the WW domain-containing transcription regulator 1 (*Wwtr1*) gene.^90^ This decrease in Wwtr1, which works in association with YAP in mechano-transduction signaling, is proposed to reflect the changes in perivascular spreading of pericytes with the introduction of cancer cells.^90^

There are conflicting results on the effect of pericytes on cancer cell proliferation. Brain pericytes secrete insulin-like growth factor 2 (IGF-2) to stimulate the proliferation of mammary carcinoma cells, but not melanoma cells, and blocking IGF-2 decreases the BrM size in the breast cancer mouse model.^73^ Another experiment, albeit focusing more on lung metastasis, showed that co-culturing aggressive breast cancer cells with primary lung pericytes led to more persistent disseminated tumor cells in the brain and lung when compared to cancer cell monoculture.^91^ The mechanism of enhanced lung metastasis is through promotion of cancer dormancy by exposure of tumor cells to lyso-phospholipid from tumor-cell-activated pericytes.^91^ This pericyte-activated cancer dormancy is suggested to be relevant in the brain,^91^ but further studies are needed to confirm the hypothesis.

Alternatively, the growth-suppressing effect of pericytes on cancer cells has also been described. Either co-culturing with pericytes or applying pericyte-conditioned medium inhibited colonization of the brain by the brain-metastatic variant of human squamous cell lung carcinoma.^74^ This result was replicated in another *in vitro* BBB model study showing an inhibition of proliferation of brain-tropic bronchial squamous cell carcinoma by pericytes.^90^ Thus, further studies with in-depth mechanistic exploration are needed to demonstrate the effect of pericytes on metastasizing cell proliferation.

### Pericyte Involvement in the Formation of Brain Macrometastasis and TME

**Figure 4.**
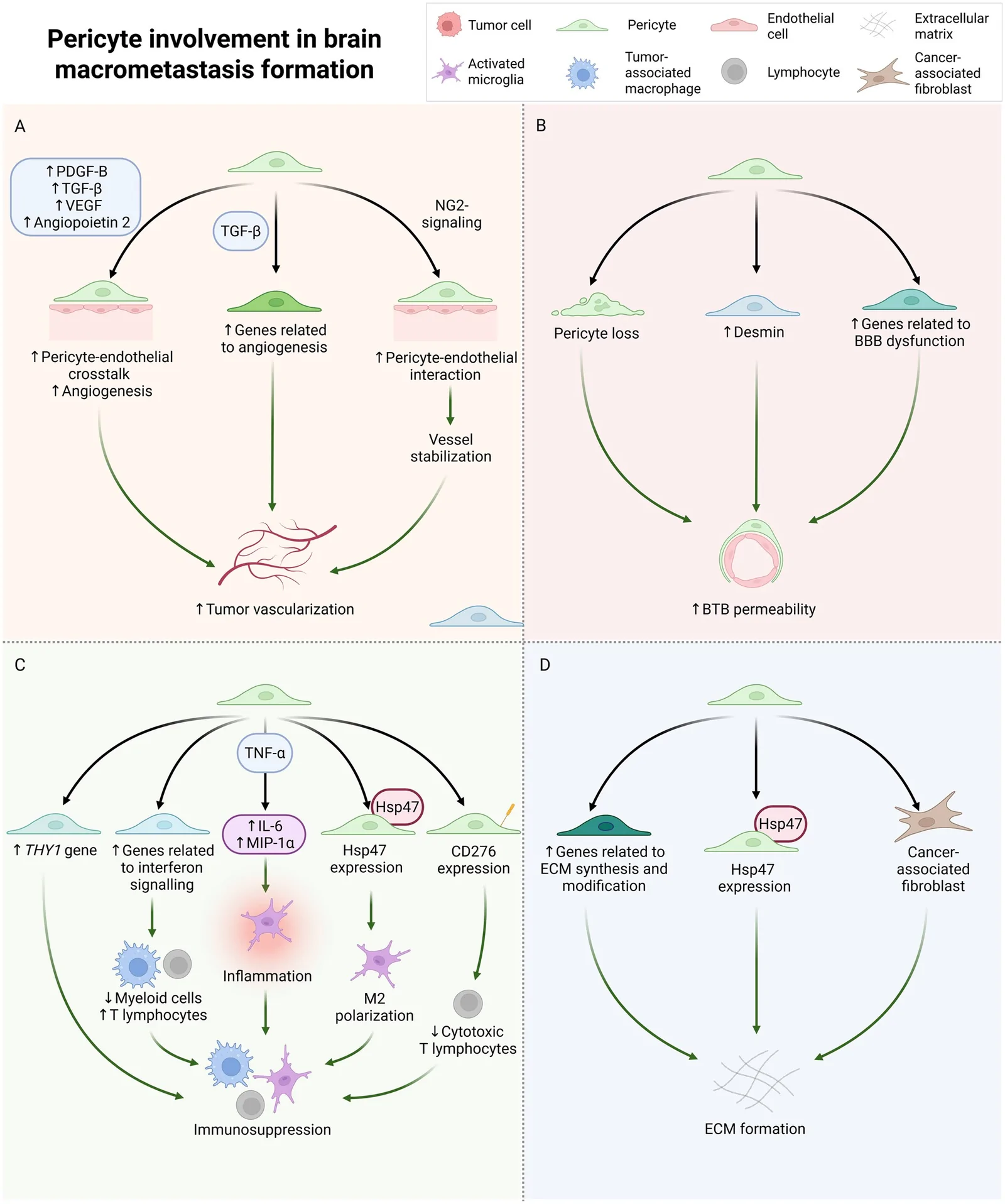
Pericyte involvement in brain macrometastasis formation. (A) Pericyte enhances vascularization in brain metastases. Increased platelet-derived growth factor subunit-B (PDGF-B), transforming growth factor beta (TGF-β), vascular endothelial growth factor (VEGF), and angiopoietin 2 within the tumor microenvironment enhance pericyte-endothelial crosstalk, thereby promoting angiogenesis. TGF-β also enhances pericyte expression of genes related to angiogenesis. The proteoglycan neuron-glial antigen 2 (NG2) on pericytes participates in pericyte-endothelial interactions, thereby promoting proper tumor vessel stabilization and vascularization. (B) Increased blood-tumor barrier (BTB) permeability in brain metastases is the result of pericyte loss, increased pericyte desmin expression, and increased gene expression related to blood-brain barrier (BBB) dysfunction. (C) Pericytes promote an immunosuppressive microenvironment in metastatic brain tumors via an increase in *THY1* gene expression. In addition, upregulation of pericyte genes involved in interferon signaling alters the immune cell composition of the tumor microenvironment by decreasing myeloid cells and increasing T-lymphocytes. Tumor necrosis factor alpha (TNF-α) induces pericyte secretion of macrophage inflammatory protein (MIP)-1α and interleukin (IL)-1α, thereby causing inflammation. Heat shock protein-47 (HSP-47) on pericytes can drive microglial M2 polarization, leading to immunosuppression. Brain pericytes also express CD276, leading to reduced cytotoxic T-lymphocyte populations and an immunosuppressive environment. (D) Brain pericytes contribute to extracellular matrix (ECM) formation in brain metastases. The upregulation of genes related to ECM synthesis and modification in these mural cells, pericyte expression of heat shock protein 47 (HSP-47), and the development of cancer-associated fibroblasts are involved in this process. Created in BioRender. Hirunpattarasilp, C. (2026) https://BioRender.com/kj6etsm.

In TME, there is an alteration of the number and protein expressions of pericytes, which can affect their roles in tumor vascularization, BTB maintenance, immunomodulation, and ECM formation (Figure 4).

#### Changes in pericyte number and expression in TME

Most studies found a loss of approximately 23%-75% of resident pericytes in BrM vessels.^39^^,^^46^^,^^87^ However, one study found an increased ratio of PDGFR-β+ pericytes to CD31+ endothelial cells in human breast and lung BrMs without increasing vessel coverage.^75^ Pericyte loss could be from excess PDGF-B,^42^ VEGF,^61^^,^^63^ and angiopoietin 2 signaling in the TME (Table 1).^42^^,^^66^ A recent transcriptomic study also identified class 3 semaphorin (SEMA-3) in astrocytes and endothelial cells as a key regulator of BrM vascular permeability.^63^ Although the study did not specify pathways in pericytes, SEMA-3 can inhibit cerebral pericyte recruitment by disrupting PDGF crosstalk between pericytes and endothelial cells.^92^

To our knowledge, no study has investigated whether the continuum of pericyte phenotypes along the microvasculature is preserved in BrMs. However, the architecture of the microvasculature is altered by the abnormal tumor vascularization,^16^^,^^35^^,^^36^ and the arteriovenous zonation of BrM vasculature based on gene signatures of endothelial populations is markedly different from that of the normal brain, with only 36% of cells able to be mapped to the arteriovenous axis.^75^ Thus, it is likely that the continuum of pericyte phenotypes is affected. Transcriptomic analysis also supports that BrM pericytes are distinct from normal brain pericytes.^75^

#### Tumor vascularization and metabolic reprogramming

Alterations in pericytes and disrupted pericyte/endothelial crosstalk through PDGF-B, TGF-β, VEGF, and angiopoietin 2 in the TME have been shown to stimulate angiogenesis and growth of BrMs (Table 1).^42^^,^^58^^,^^59^^,^^64^ In lung BrMs, tumor angiogenesis is triggered by enhanced VEGF signaling. This VEGF pathway is stimulated by CD146+ pericyte-like cells, derived from CD44+ lung cancer stem cells, which interact with reactive astrocyte-derived growth arrest-specific 6 (GAS6) to overexpress CD146 and assume pericyte-like morphology.^64^^,^^93^ Consequently, targeting CD146 or GAS6 receptors exerted anti-angiogenic effects on BrMs *in vivo*.^64^ Pericyte NG2 also participates in the proper vascularization of BrMs because NG2-null and pericyte-specific NG2-ablated mice, intracranially injected with melanoma cells, showed abnormal tumor vascularization and retarded melanoma BrM growth.^67^^,^^94^ These vessels had reduced pericyte coverage and impaired pericyte/endothelial cell interactions, leading to disruption of pericyte maturation and collagen deposition in the basement membrane.^67^^,^^94^ This led to decreased patency and increased leakiness in vessels, ultimately resulting in tumor hypoxia.^67^^,^^94^

Transcriptomic analyses of human BrM pericytes also show increased angiogenic gene signatures.^59^^,^^75^ RNA sequencing analyses have identified a “proliferative” pericyte cluster, expressing genes related to angiogenesis and plasticity, cell cycle progression, and protein translation, that was not found in brain pericytes from the non-tumor tissue.^75^ The role of pericytes in tumor angiogenesis is further supported by another study of RNA transcriptomic analysis of human BrM pericytes, demonstrating an activation of angiogenesis and epithelial-mesenchymal transition signaling pathways as a result of the interaction of pericytes with TGF-β released by various cells in the TME.^59^

Unique pericyte changes are found in co-opted vessels incorporated into BrMs, depending on the tumor growth type. In human BrM tissue from various primary tumors, a duplication of the PDGFR-β+ pericyte layers with a high amount of collagen was found within the vessels at the surface of the pushing-type BrMs, and less frequently, inside the papillary-type tumors.^52^ The mural cells in the inner layers were consistently positive for α-SMA, suggesting a role in blood flow regulation, and pericytes in both layers expressed heat shock protein-47 (HSP-47), which participates in collagen biosynthesis.^52^ This double layer of pericytes is thought to result from increased pressure exerted by the BrMs on normal brain parenchyma. During the process of vessel co-option at the tumor-parenchymal interface, pushing-type tumors separated the outer pericyte layer of the vessels. They assimilated only the inner pericyte layer and endothelial cells into the tumor, leaving the outer pericyte layer along the tumor-parenchymal interface. In contrast, the papillary-type tumors assimilated vessels with both layers of pericytes.^52^ The role of pericytes in this process remains to be elucidated. However, these mural cells participate in ECM formation in both BrM types (see below).^52^ There is no data on pericyte involvement in BrM vascular mimicry, but there is evidence that they assist in stabilization of these vessels in other tumors, including primary brain tumors.^71^^,^^95^

These studies demonstrate the importance of brain pericytes in tumor vascularization. Because access to oxygen and nutrients governs TME formation and the metabolic reprogramming of cancer cells,^40^ pericytes are among the metabolic participants in the TME.

#### BTB maintenance

Alteration of pericytes in the BrM contributes to the increased permeability of the BTB. The reduction of pericyte number (see above) is related to elevated permeability of the BTB, because inducible deletion of pericytes in a mouse breast-BrM model downregulated claudin-5 and increased BTB leakage.^75^ TGF-β in the TME can also cause pericyte metabolic changes and disrupts the barrier.^60^

Another contributing factor to BTB “leakiness” is the change in pericyte subpopulations, particularly the expansion of desmin+ pericytes.^39^^,^^45^^,^^46^ Increased pericyte desmin expression was found in mouse BrMs of non-small cell lung cancer and breast origins,^39^^,^^45^^,^^46^ and the development of desmin+ pericytes was related to elevated BTB permeability.^45^ In human tissue, one study showed inconsistent desmin expression in pericytes in non-small cell lung cancer-BrMs,^39^ but the association of desmin+ pericytes and permeability in BrMs of breast and lung origins was confirmed in another study.^46^ In comparison, the change in CD13 in pericytes in the BTB is inconclusive. One study of a mouse BrM model of non-small cell lung cancer found an increase in pericyte CD13 expression,^39^ while another demonstrated a reduction of this marker in BrMs from a mouse breast cancer model and human specimens.^46^

Increased BTB permeability is also reflected in changes in pericyte RNA expression. Bulk RNA sequencing of human BrM pericytes and endothelial cells noted an upregulation of the “BBB dysfunction” module, which signifies common vascular changes found in multiple CNS diseases, and a downregulation of pathways related to BBB homeostasis.^75^

Because pericytes maintain the barrier,^75^ they represent a therapeutic target to increase BTB permeability and allow greater penetration of chemotherapy. Nanoparticles targeting intercellular adhesion molecule 1 (ICAM-1) were developed to deliver drugs specifically to BTB endothelial cells and pericytes.^96^ These nanoparticles were loaded with nitazoxanide to inhibit endothelial WNT signaling and ibrutinib to target bone marrow and X-linked nonreceptor tyrosine kinase (BMX), which is exclusively expressed by pericytes and is vital in the maintenance of these cells. Nitazoxanide and ibrutinib co-loaded ICAM-1-targeting nanoparticles were effective in opening the BTB without affecting normal brain vessels and were able to improve chemotherapeutic efficiency against BrMs.^96^

#### Immunomodulation

Changes in BrM pericytes shape TME by interacting with immune cells and promoting an immunosuppressive environment.

Single-cell RNA sequencing analysis of human BrM pericytes showed enrichment in the *THY1* gene,^75^ which is associated with immunosuppression in malignant gliomas.^97^ Another single-cell transcriptomic study of breast cancer also showed that pericytes at metastatic sites (including BrMs) had upregulation of the *THY1* gene compared to those at the primary site.^98^ Moreover, RNA sequencing of human BrM pericytes identified an “interferon” pericyte cluster, that expressed signatures of antigen presentation and interferon signaling.^75^ This pericyte cluster was associated with fewer myeloid cell proportions, but more T-cell populations in BrMs. Furthermore, inducible deletion of pericytes in a breast-BrM mouse model increased the total immune cells and tumor-associated macrophages (TAMs),^75^ supporting the role of BrM pericytes in immunosuppression.

Single-cell RNA sequencing and bulk analysis of human lung-BrMs showed that TGF-β signaling between pericytes and other cells within the TME led to the formation of unstructured BrM vasculature, which helps shape immunosuppressive environments.^59^ Moreover, increased TNF-α within BrMs could trigger pericyte release of inflammatory cytokines, such as IL-6 and MIP-1α, to activate and transform BrM-associated microglia to assume stellate and reactive amebic morphology.^28^^,^^58^^,^^81^

HSP-47+ pericytes were found in incorporated vessels in human BrMs,^52^ and HSP-47 stimulates microglial M2 polarization through the HSP-47-collagen axis.^50^ Interestingly, blocking the pathway restored the anti-tumor immunity and enhanced the treatment effect of immunotherapy in BrMs.^50^

Pericytes also modulate the BrM immune landscape via immune checkpoint molecules. BrM pericytes from both human and mouse models show an overexpression of CD276, which is an immune checkpoint molecule that suppresses immune function.^75^ Blocking CD276 with antibodies in the mouse breast-BrM model increased survival, decreased tumor growth, and increased infiltration of cytotoxic T cells.^75^ In addition, another study using machine learning models identified five pericyte-related genes (*PEA15*, *CCZ1*, *HMGB1*, *RPL29,* and *SPRY4*) that predict responses to immune checkpoint inhibitor therapy in lung-BrM patients, suggesting these genes could represent targets to improve the efficacy of immunotherapy.^59^

In cancers of other organs, pericytes promote macrophage M2 polarization and infiltration through CXCL14 and lactadherin pathways.^40^ TAMs, in turn, secrete cytokines to stimulate pericyte recruitment and angiogenesis.^40^ Further investigation of these pericyte-macrophage interactions in BrMs might provide new information on the immunosuppressive role of BrM pericytes.

#### ECM formation and development of CAFs

BrM pericytes contribute to ECM formation. As previously mentioned, during vessel incorporation, pushing-type BrMs retain the HSP-47+ pericytes at the tumor surface, while papillary-type BrMs incorporate these cells.^52^ The HSP-47+ pericytes are the main source of connective tissue in BrMs, causing collagen accumulation at the surface of the pushing-type BrMs and in the center of the papillary-type metastases.^52^ Moreover, RNA sequencing analysis of pericytes from human BrMs of lung and breast origins identified an “ECM” pericyte cluster, expressing gene signatures related to extracellular matrix synthesis and modification.^75^ Thus, these analyses highlight the role of pericytes in ECM formation in BrMs.

Moreover, CAFs can derive from pericytes. In human BrMs from various primary tumors, CAFs are connected to endothelial cells and pericytes and could co-express α-SMA and PDGFR-β, which are pericyte markers.^51^ Furthermore, pseudotime analysis of single-cell RNA sequencing of human BrMs shows that both inflammatory-like and myofibroblast CAFs originated from the same pericytes.^59^

## Future Directions and Clinical Implications

### Outstanding Questions

Studies on brain pericytes in BrMs are limited, despite their vital roles in BrM formation and maintenance. To further understand this multifaceted role of pericytes in the complex BrM process and develop therapeutic approaches targeting these cells, further investigation is vital, especially to address the following topics:

#### Origin of pericytes in BrMs

The origin of pericytes in BrMs remains poorly understood. Evidence has shown the incorporation of cerebral blood vessels containing resident pericytes^52^; however, lung cancer stem cells can also assume pericyte-like morphology in BrMs.^64^^,^^93^ Intravital microscopy to study the development of BrMs in real-time and pseudotime analysis of RNA sequencing may help clarify the source of BrM pericytes. This knowledge would provide an opportunity to intercept BrM pericytes development.

#### Specific markers for BrM pericytes

BrM pericytes have altered gene expression and proteins when compared to those in normal brain tissue.^75^ Further investigations into the specific markers of BrM pericytes that differentiate them from those in the brain would be ideal for identification and creating treatment aiming specifically at BrM pericytes.

#### Heterogeneity among BrMs of different origins

Pericytes in BrMs from different primary origins might be heter­ogeneous.^73^^,^^75^^,^^77^ There are a few studies that compared BrM pericytes from different metastatic primaries.^52^^,^^99^ Further investigation, including transcriptomic and proteomic studies, comparing BrMs from different primary types, would provide a better understanding of pericyte function in each tumor type and aid in identifying treatments for the specific origin of BrMs.

### Clinical Implications

Currently, there is an immense need for new BrM treatments. Brain pericytes could be a promising therapeutic target due to their roles in BrM formation and maintenance, and there are promising data from preclinical studies.

#### Targeting pericytes for prevention of BrM formation

Effective prevention strategies for BrM are being investigated.^10^ PlGF, which could act on pericytes to promote PMN formation,^61^^,^^78^ might be an interesting therapeutic target. Inhibition of COX-2, TNF-α, and CCL/CXCL should also be studied to prevent PMN formation.

#### Targeting pericytes to interrupt micrometastasis formation

Pericyte-derived IGF-2 has pro-tumorigenic effects, and inhibition of IGF-2 leads to decreased BrM size.^73^ Pericyte-secreted IGF-2 could be a potential target for BrM treatment.

#### Targeting pericytes to reduce tumor vascularization

Crosstalk between pericytes and endothelial cells, including PDGF, VEGF, and angiopoietin pathways (Table 1), is a potential therapeutic target for inhibiting tumor vascularization. Moreover, NG2-null mice exhibit impaired tumor vessels,^67^^,^^94^ and inhibition of CD146 with imaprelimab has anti-angiogenic effects on BrMs,^64^ raising the possibility of using NG2 and CD146 blockade as BrM treatments.

#### Targeting pericytes to increase chemotherapy penetration

Nitazoxanide- and ibrutinib-loaded ICAM-1-targeting nanoparticles act on pericytes and endothelial cells to open the BTB, thereby enabling greater CNS penetration of chemotherapy in a mouse model.^96^ This technology might be beneficial in increasing chemotherapy penetration in BrMs.

#### Targeting pericytes for immunomodulation

Another interesting approach is blocking CD276 on BrM pericytes to promote anti-tumor immune responses, as antibodies to CD276 increased survival and reduced tumor growth in a mouse BrM model.^75^ Moreover, five pericyte-related genes have been identified to predict responses to immune checkpoint inhibitor therapy,^59^ representing an opportunity to check these genes to identify patients who would be more likely to respond to immunotherapy. Furthermore, inhibiting HSP-47+ pericytes might help reduce collagen synthesis and restore anti-tumor response in BrMs.^50^

Despite the potential for developing brain-pericyte-based therapeutic approaches, several challenges remain to be addressed. Firstly, there is limited data in humans. Testing these therapeutic modalities in human tissue and patient-derived organotypic culture would be required before developing clinical trials. The second obstacle is the off-tissue effect. Pericytes are present throughout the body, and there is no specific marker for them, raising the possibility that they affect other cells. Despite this limitation, the development of pericyte-targeting drug delivery is currently under investigation.^100^ Lastly, therapeutic approaches need to achieve good CNS penetration given the intracranial location of cerebral pericytes, further complicating the development of treatments. Once these are addressed, pericyte-targeted approaches could represent a new intervention for BrMs.

## Conclusions

In this review, we have shown that brain pericytes participate in both promoting and inhibiting BrM formation and maintenance. Further studies are needed to better understand how these cells engage in BrMs, providing more insights into the mechanisms of BrM colonization and formation, and helping develop therapeutic approaches. An interdisciplinary effort to combine methods, ranging from cell cultures to more complicated preclinical studies, is vital to study both the spatial and temporal dimensions of BrMs. Another key investigation is the translation of results from animal studies to clinical trials. With all things considered, pericytes play various roles in brain physiology and participate in multiple steps of BrM formation, making brain pericytes an attractive therapeutic target for the treatment of BrMs.
